# A critical synthesis of the role of the pharmacist in oral healthcare and management of medication-related osteonecrosis of the jaw

**DOI:** 10.1038/s41405-020-0043-7

**Published:** 2020-08-11

**Authors:** A. Sturrock, P. M. Preshaw, C. Hayes, S. Wilkes

**Affiliations:** 1 Faculty of Health Sciences and Wellbeing, The Sciences Complex, University of Sunderland, City Campus, Chester Road, Sunderland, SR1 3SD UK; 2 National University Centre for Oral Health, National University of Singapore, Singapore, Singapore; 3 Newcastle University, Newcastle upon Tyne, UK; 4 Universities of Cumbria and Liverpool Hope, Liverpool, UK

**Keywords:** Osteonecrosis of the jaw, Oral hygiene

## Abstract

**Objective:**

To consolidate extant published evidence in relation to the potential of integrating oral healthcare for patients at risk of developing medication-related osteonecrosis of the jaw (MRONJ).

**Methods:**

A critical synthesis and consolidation of five publications was undertaken. As a mechanism of situating the extant work within the context of primary healthcare provision, the Rainbow Model of Integrated Care was applied as a theoretical lens through which the conceptual findings could be collectively applied to practice.

**Results:**

The critical synthesis revealed a thematic emergence relating to both formative and normative integration. The most salient of these were the identification of limited shared clinical records, and disconnection of oral healthcare provision from patients’ general medical care. The three levels of the Rainbow Model of Integrated Care reflected a series of issues for address.

**Conclusion:**

In the context of collaborative, multi-disciplinary working for patients at risk of development of MRONJ, pharmacists are a professional group which this research reveals to be an underutilised resource. Reduction of oral health inequality at all levels of patient care is a key priority and this research highlights areas for address in relation to requirements for interprofessional education, optimal communication and policies reflective and facilitative of these.

## Introduction

The provision of preventive oral healthcare advice has traditionally been the preserve of dental health professionals; a situation that has been established over the years as a result of the differing training, clinical practice and healthcare delivery systems that exist between dental care and other aspects of healthcare. However, over recent years there has been increasing recognition of the links between oral health and a number of systemic diseases and conditions, as exemplified by the relationships that have been identified between periodontitis and cardiovascular disease and, most notably, type 2 diabetes.^1–4^

Wilson and Soni, the former presidents of the British Dental Association and Royal Pharmaceutical Society, respectively, in 2016 published an opinion piece in the *British Dental Journal*; emphasising the opportunities for pharmacy and dentistry to spearhead a new era of interprofessional healthcare.^5^ The existing literature highlights a potential role for pharmacists in the provision of oral healthcare; a number of studies have identified that patients already attend pharmacies for advice in relation to oral health concerns and that pharmacists agree that the provision of oral healthcare is part of their role.^6–8^ However, studies have demonstrated that pharmacists have limited awareness of the links between oral and systemic health.^9^ There is also minimal existing published literature exploring the relationship and integration between pharmacy and dental service providers, with evidence highlighting a lack of communication and collaboration, despite there being a positive interest in developing collaborative relationships.^10,11^

There is also a lack of published literature on the role of pharmacists in relation to the oral health-related adverse effects of medications. One such condition is medication-related osteonecrosis of the jaw (MRONJ), which represents a significant adverse effect of a range of anti-resorptive and anti-angiogenic drugs, such as the bisphosphonates and denosumab. MRONJ is a complex phenomenon and due to the significant morbidity of the condition and the limited treatment options available for its address, the elimination or stabilisation of oral disease before initiating implicated medications is recommended as a preventative measure.^12^

MRONJ is relatively rare, the estimated incidence of MRONJ in cancer patients treated with anti-resorptive or anti-angiogenic drugs is 1% and in osteoporosis patients treated with anti-resorptive drugs is 0.01–0.1%.^12^ Therefore, whereas pharmacists are unlikely to encounter patients with MRONJ regularly in practice, they do have close and frequent contact with patients prescribed implicated medications.

Current guidelines published by the Scottish Dental Clinical Effectiveness Programme recommend an interprofessional approach to the prevention of MRONJ.^12^ This clearly represents an opportunity for pharmacists, in all settings, to engage with this patient group and provide education to patients and other members of the interprofessional healthcare team on the risk of the development of MRONJ and appropriate preventive measures.

Accordingly, in this study, we sought to investigate the integration of oral health promotion and advice regarding the prevention of MRONJ into the role of pharmacists.

## Methods

A series of five independent studies were undertaken by the authors of this paper.^13–17^ This body of published work contributes to the investigation of MRONJ, and the role of pharmacists in both MRONJ prevention and oral health promotion; which are fields that remain largely underreported in the extant literature. In this paper we present a critical synthesis of these five previously published papers, which substantively integrates the findings of the published work. This was undertaken via a theoretical lens which was conceptually appropriate to the context of care, the Rainbow Model for Integrated Care.^18^

Three of the published studies explored the interprofessional prevention of MRONJ through qualitative semi-structured interviews with pharmacists,^13^ general medical practitioners (GPs),^13^ general dental practitioners (GDPs)^15^ and patients.^14^ The fourth study explored the integration of oral healthcare advice into the role of clinical pharmacists working in general medical practice.^16^ An interpretive qualitative approach was adopted in each of these four studies, with constant comparative analysis facilitating an iterative process of data collection and analysis.^19^ Semi-structured interviews and focus groups were transcribed verbatim and framework analysis facilitated the prioritisation of salient themes.^20^ A total of 77 participants were interviewed across these four studies; 19 pharmacists, 11 GPs, 15 GDPs, 29 patients, 2 nurses and 1 practice manager. The fifth study piloted the provision of a brief oral health promotion intervention to 1069 patients in community pharmacies in County Durham, UK.^17^

The Rainbow Model of Integrated Care was originally designed to facilitate clinicians and researchers in achieving a better understanding of the concept of integrated care from a primary care perspective.^18^ It has been applied to a range of studies, including qualitative and survey-based research and systematic reviews.^21–24^ This conceptual framework was adopted in this study as a theoretical lens through which the barriers to, and facilitators of, integrating MRONJ prevention and oral healthcare into the role of the pharmacist could be explored in practice. Themes from each individual study were used as a mechanism of collective guidance in the initial critical synthesis. During this process, the original raw data in the form of verbatim transcripts were collectively revisited and reanalysed via the theoretical lens of the Rainbow Model of Integrated Care.

Central to the model is the co-ordination of concepts into three dimensions that capture the individual complexity and interdependent elements of collaborative healthcare alongside the various forms of integration required for optimal levels of person-centred and population-focused healthcare.

*Macro: external structural, social and regulatory issues which are beyond the control of the individual or influence of individual organisations.*

*Meso: local community and institutional factors and influences.*

*Micro: day-to-day practices of individuals and their practice environments.*

Person-focused healthcare is based around the premise that diseases are simultaneously medical, psychological and social problems, with mechanisms of care provision based on personal preferences, needs and values. Population-based healthcare indicates that services should be designed and delivered in line with the needs and characteristics of a defined population as well as their expressed preferences.^18^

**Fig. 1:**
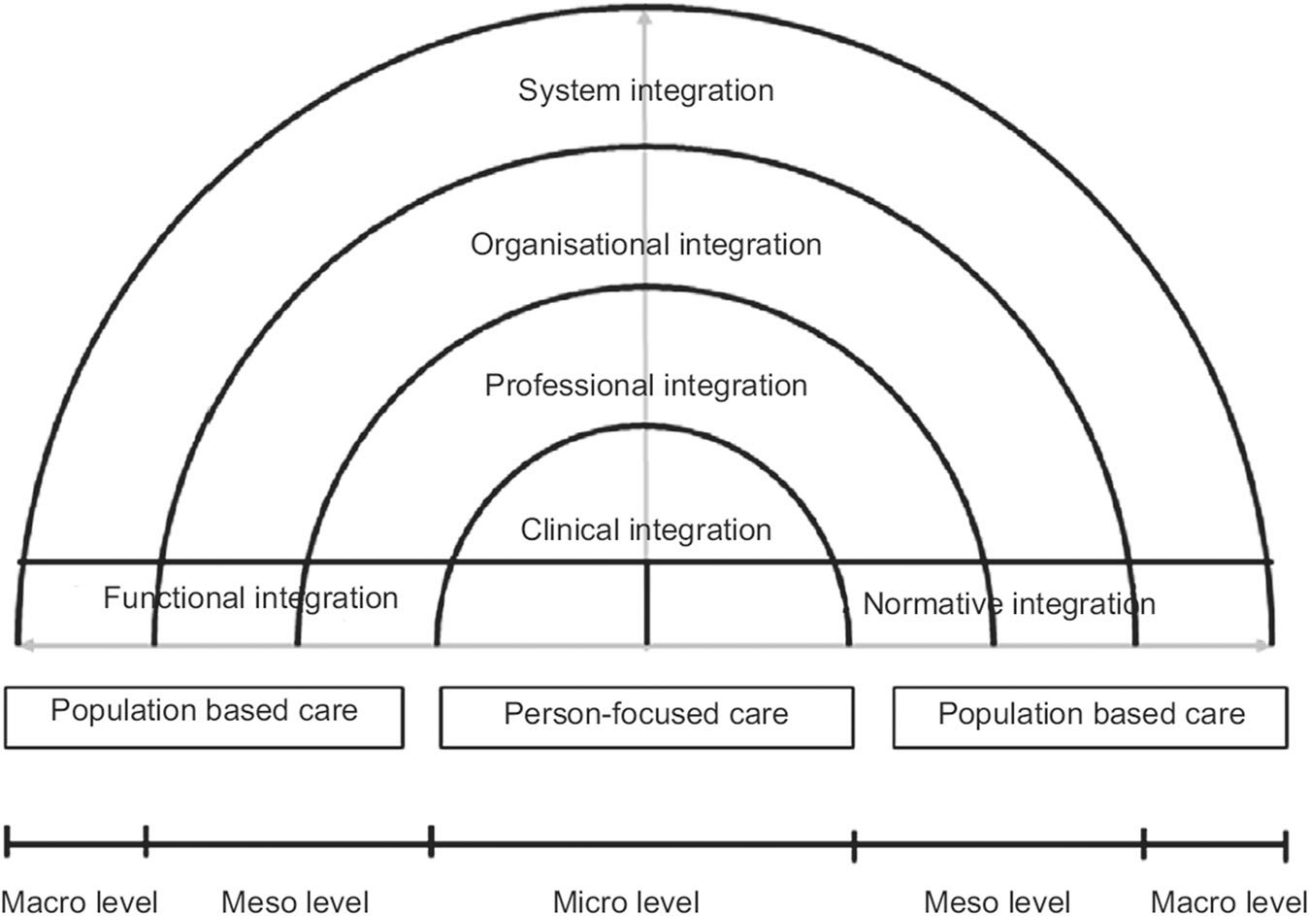
Rainbow Model of Integrated Care.^18^ The model is a means of visually framing the concept of clinical integration, in the context of patient centred care. The liminal levels represented at macro, meso and micro levels provide an insight into the population-based approaches adopted and how these are effectively translated into practice at macro and, consequently, micro levels.

The Rainbow Model of Integrated Care was produced as a means of visually conceptualising each of these components of integrated care (Fig. 1). Central to the model is the individual patient, visualised through the concept of patient-focused care and processes of clinical integration. At a meso level, both professional and organisational integration emphasise a population-based approach. At a macro level, systems integration places the patient at the centre of the healthcare system, embracing the premise that what is best for the individuals within a defined population is also best for the population as a whole.^18^ Both vertical and horizontal integration are fundamental components of systems integration. Vertical integration includes strategies which link the various levels and degrees of specialised care within and between sectors; this includes integration of services from a primary care perspective, with both secondary and tertiary care settings. Horizontal integration takes a more holistic view and includes strategies which link similar levels of care across sectors, and acts to improve the health of individual patients and populations.^18^

Valentijn et al.^18^ describe a range of other forms of integration that are required for effective collaborative and integrated care at the meso (organisational and professional integration) and micro level (clinical integration). Functional and normative integration also link the micro, meso and macro dimensions of the healthcare system.^18^

### Ethics

Formal ethical approval for each of the studies selected for inclusion in this critical synthesis had previously been granted by the University of Sunderland Ethics Committee.

## Results

A range of barriers to, and facilitators of, MRONJ prevention and oral healthcare integration into the role of the pharmacist was identified and aligned to the constructs of the Rainbow Model for Integrated Care. Direct qualitative quotes from verbatim transcripts of interviews are included in Table 1 as a means of illustrating the integration of findings from across the original research studies undertaken.

**Table 1:** Themes and supporting qualitative quotes.

Theme	Quote (Q) reference	Quote
NHS policy and dental care services	Q1	The processes of how you get people to take their dental health seriously are very difficult. The ones that pay for dentistry are likely to be the ones with good teeth, the others who get free treatment just don’t access it. (GP3)^13^
	Q2	The area I am in is very deprived and actually, I would say that the majority don’t ever visit the dentist, I think they just don’t see it as important and loads of them just don’t have the money, and fear, loads of people hate seeing a dentist unless it’s absolutely necessary. (Pharmacist 5)^16^
Education of patients and preconceptions	Q3	I think it [oral health education] would be from my mum and dad and then the dentist. I don’t think anyone else has ever talked about oral health with me, maybe the school nurse a long time ago. (Patient 5)^16^
	Q4	I think it’s just the way society has brought us up in that the, there are two defining people, dentists and doctors. Anything to do with dentists, you go to the dentist. Anything about your health you go to the doctors. They have always been seen as separate. (Patient 6)^16^
Isolation	Q5	I think with a lot of things with dentists really, that we are out of the loop, I just don’t seem to have had much interaction with any other healthcare professionals. (Dentist 6)^15^
Communication and collaboration	Q6	I’m directly contactable face-to-face by prescribers, GPs, nurse practitioners, nurses, admin team, everything. They can just come directly into my office and ask me for information. So, I’m probably more likely to be utilised clinically. (Pharmacist 1)^16^
Local population needs	Q7	In that 2 min that you have got to hand something out to somebody, you concentrate on the important things, such as how to take it, to get their concordance and compliance. (Pharmacist 2)^13^
Education of healthcare professionals	Q8	I personally don’t really feel that I’ve got a good enough understanding of what an actual pharmacist’s job entails. (Dentist 2)^15^
	Q9	No not really… [we had] a little bit [of shared education] in lectures maybe at university but not with dentists. We have worked quite closely with the doctors but not with dentists. (Pharmacist 1)^13^
Practitioner’s competence and capability	Q10	I’m in quite an advanced clinical role now. So I do a lot of diagnostics and treating myself. I’m a prolific prescriber. (Pharmacist 7)^16^
Professional hierarchies	Q11	My clinics could easily be timetabled for 20 min instead of 10, and as I don’t really see acute patients or have the same time pressures as some of the GPs or practice nurses. I can talk longer and to go into more detail about things, there is scope to take more time and really reinforce the key messages. (Pharmacist 2)^16^
Individual clinical competencies	Q12	I think most of us know the basics, but not really much depth, especially around how oral health and just general health and wellbeing are related. (Pharmacist 3)^16^
	Q13	It’s the sort of thing that once you see it, you then remember it [MRONJ]. They were both very complex patients, but the amount of morbidity involved with the osteonecrosis of the jaw in both of those patients was considerable. (GP 1)^13^
Professional relationships	Q14	I feel as though the pharmacist that I go to, I could ask her anything and she would tell us. I have had a review with her, she’s very, very helpful and knowledgeable about medication. (Patient 5)^15^
	Q15	If you have to wait to get an appointment with the pharmacist at the doctor’s surgery, you may as well just see the doctor or whatever else. The point of a pharmacist to me it that it’s, like, around the corner and it’s easy. (Patient 6)^16^
Isolated working patterns	Q16	We don’t seem to engage with dentists. In fact, the only time that I ever had a proper conversation with a dentist was when I worked in community pharmacy and that would have been over an incorrect prescription or an out of stock item. And I just think, you know, there is a lot of cross-conversations that we could have. (Pharmacist 10)^16^
	Q17	I make sure I take medication histories for patients, but they don’t always know exactly what they take. It’s sometimes hard to be sure the list they give you is accurate. (Dentist 15)^15^
Individual patient needs	Q18	I think counselling about medication is far better done by the pharmacists. I think the other reason is perhaps, when a patient sees a doctor they expect to be able to discuss all aspects of their lives and their care. (GP 1)^13^
	Q19	This is difficult, but mentally, it gives you some kind of anxiety because you- you- you know your bone is [visible] there- a little piece of [exposed] bone on your left-hand side is there. (MRONJ Patient 5)^14^
	Q20	Psychological and mental, yeah. If you’re going out to a restaurant, then you have to be very careful. You don’t want people to see that you are eating awkwardly. (MRONJ Patient 5)^14^
Disconnection with general health	Q21	I haven’t really heard of links between the two. I see lots of patients with diabetes and it is definitely not something that I would tell patients about. (Pharmacist 5)^16^
Lack of shared clinical records	Q22	It would be brilliant, if we could just see, even just an element of their records, even just what drugs they were taking. That’s the main thing for us, it takes so long to get the drug history out of a patient. (Dentist 13)^15^
	Q23	We would never know if the dentists had prescribed any antibiotics or anything for a patient. Yet, if anyone else in the primary healthcare team prescribes anything for our patients, we know. (Pharmacist 7)^16^
Sub-optimal referral pathways	Q24	I would say there is anonymity really [between dental and medical service providers]. If you compare it with, for example, local opticians where we have frequent interactions, albeit by paper, we don’t really get any, sort of, direct contact. Not that I can recall. (GP3)^16^

### Macro (system integration)

#### NHS policy and dental care services

Participants reported that dental care services in the UK have traditionally placed limited emphasis on preventive dental care (Quote 1, Q1); a situation that has likely arisen, at least in part, from remuneration systems that have rewarded interventions to deal with the consequences of dental diseases rather than their prevention. Although new methods of care delivery that prioritise prevention are currently being piloted in the UK, they have yet to be implemented at scale. Prevention is particularly important when considering the management of MRONJ, as good oral hygiene and preventive dental treatment can significantly reduce the risk of developing the condition.

There is an identifiable need for the system to facilitate an increased emphasis on engaging patients and other healthcare professionals with mechanisms of oral health promotion and preventive care; this relates to the education of patients and to the structure and funding of dental services. NHS dental care in England requires that the majority of adult patients must pay a proportion of the cost of their dental treatment (akin to ‘co-payments’); however, for some people on low incomes, they are exempt from all charges. The requirement for individuals to pay a proportion of the costs was described as a significant barrier by both patients and healthcare professionals to the engagement of certain sectors of the population in regular preventative dental care and intervention (Q2). Supportive and prevention-based oral healthcare policies could potentially facilitate enhanced levels of engagement of both patients and healthcare professionals in attending and facilitating oral healthcare, respectively.

#### Education of patients and preconceptions

Oral health education for patients is typically only provided in a dental setting (Q3). This strengthens the long-held assumptions of dental and medical services as being disciplinary silos, which, in turn, acts as a barrier to the full integration of dental and general healthcare. A commonly identified theme is the phobia of dental treatments reported by many patients (Q2). A move to preventive dental care to reduce the need for invasive dental treatment has the potential to engage more patients with oral healthcare and, as a consequence, enable timely intervention and advice.

There is also a need for patient education on what the roles of the healthcare team constitute; pharmacists were recognised as good sources of healthcare information, but there has historically been limited engagement with oral health advice as part of their role. The patients interviewed in this work described growing up with healthcare professionals undertaking stereotypical traditional roles, with little evidence of dental education forming part of any contact with healthcare professionals other than dentists (Q3, Q4).

In order to fully engage the public with oral health, the relative degree of abstraction and physical isolation of dental care from general health and well-being services needs to be addressed so that it can be better integrated into the interprofessional team and serve to challenge society’s often misplaced preconceptions and presuppositions about practices and processes of dental care. The positive response of patients towards our intervention piloted in County Durham found that very few patients receiving the intervention considered that a pharmacy was not an appropriate place to receive advice about their teeth (3%);^17^ demonstrating the acceptability to patients of pharmacy’s increased role in the provision of oral healthcare.

### Meso (organisational/professional integration)

#### Isolation

All professional groups interviewed in these works cited the perception of poor working relationships between dental and medical services and isolated practices, both at an individual clinical level and at an organisational and professional level (Q5). This included both the situational and geographical isolation of dental services, which is further exacerbated by limited referral pathways between the settings and a reportedly minimal degree of interprofessional education. The lack of locally commissioned collaborative services represents a clear barrier; however, our work has shown that when oral health teams and pharmacy do collaborate, positive contributions at both the levels of population and individual patient health can be achieved. Following our pilot pharmacy-based oral health intervention, 66% of patient participants stated that they definitely intended to change the way that they looked after their teeth.^17^

#### Communication and collaboration

A significant challenge for the integration of dental and general medical care is the currently limited communication and collaboration evident between these care settings. Pharmacy and medical participants reported good lines of communication between their respective services, with pharmacists increasingly contributing to more advanced clinical services in both community and general practice settings (Q6). This was facilitated through close reciprocal interprofessional education, formal referral pathways and shared access to either full medical records (in the case of general practice pharmacists) or Summary Care Records. Such lines of communication and collaboration between primary care dental services and pharmacy/medical services do not exist, posing a significant barrier for the integration of oral healthcare across care settings.

#### Local population needs

As a meso level there are local population issues/demands that impact on the provision of services in the area. The pharmacy-based oral health intervention was piloted in parts of County Durham, which has high degrees of both social and economic deprivation. This study identified that 20% of patients had not visited their dentist in the previous 2 years.^17^ This represents an opportunity for pharmacy to engage with patients that are poor dental attenders. Many pharmacist participants did, however, report significant issues in relation to their workloads, both at an individual and institutional level. Therefore, the addition of new services and expansion of roles needs to be considered in relation to the potential of building capacity and capability for this level of potential interprofessional provision (Q7).

#### Education of healthcare professionals

Pharmacy professionals had a sound understanding of both their current and potential future role in oral healthcare provision. However, dental participants in particular had a limited understanding of what the pharmacist’s role involved, beyond a transactional approach to the supply of medication in community pharmacies (Q8). A fundamental consideration in relation to the provision of safe and effective oral healthcare is the limited degree of interprofessional education within the context of oral health advice. Participants reported limited interprofessional training with dentists at an undergraduate level and in practice, resulting in a poor understanding of the other professionals’ roles and responsibilities, and consequently a limited potential to improve holistic patient care (Q9). As a result, the scope of oral healthcare and the general competence of individual non-dental professionals in the provision of oral healthcare is exceptionally limited. Interprofessional oral health education is clearly also a system (macro) issue and needs to be an integral part of training programmes in order to develop both subject-specific knowledge and a greater capacity for more effective reciprocal and interprofessional collaborations.

#### Practitioner’s competence and capability

Although not specific to oral health-related conditions, there is clearly an advancing role for pharmacists in the delivery of clinical interventions in primary care. Many of the pharmacists, particularly those working in a general practice setting, were either working towards, or already acting as, independent prescribers (Q10). The varying clinical capability of pharmacists represents differences in the clinical responsibility and accountability in this professional group, and therefore the limitations on the care individuals provide at a micro/clinical level. The roles of individual staff and their potential to engage in oral healthcare could potentially be enhanced through upskilling the pharmacy workforce and by further education. This enhanced role represents an opportunity for pharmacists, who are the medication experts in the interprofessional team, to take greater responsibility for safe and effective medication use; this aligns with the safe prescribing and monitoring of MRONJ-implicated medicines and greater involvement with conditions such as type 2 diabetes as part of chronic disease management.

#### Professional hierarchies

Despite the increasing responsibility and accountability of pharmacy professionals identified above, there remains a perceived hierarchy from both professionals and patients, in terms of roles and responsibilities. Pharmacists would typically report signposting patients for further investigation with dental and medical issues to either the GDP or GP, respectively. This clearly represents an appropriate safety net allowing professionals to practice within the scope of their disciplinary practice and competence; however, pharmacists identified that they could potentially contribute more and in many cases are the most appropriate and accessible professional group to manage simple oral health issues in the context of primary care (Q11).

### Micro (practice integration)

#### Individual clinical competencies

The limited interprofessional education on oral health translates at a micro level to a lack of integration of oral healthcare into the current role of the pharmacist. Knowledge of oral health issues varied between individual participants, although most had a workable understanding of basic oral health promotion advice (Q12). Participants identified that a pharmacist’s role includes the promotion of healthy lifestyles, the provision of preventive advice and the safe use of medication. However, there were clear gaps in the knowledge of appropriate preventive strategies for MRONJ in most participants, and in the wider associations of oral health with general health and well-being.

Although very few participants had encountered a patient with MRONJ, the significant quality of life implications associated with the condition resulted in these individual practitioners being more vigilant towards the prevention of the condition and the education of patients at risk of its development (Q13).

#### Professional relationships

The relationships between pharmacists and both their patients and other healthcare professionals directly influence their individual practices. Both pharmacists and patients reported good reciprocal relationships, with patients highlighting the ease of access and approachability of their pharmacist, making them a valued source of health advice (Q14). Patient participants displayed a positive attitude towards the widening role of pharmacists and the integration of pharmacists into the general practice team. There were, however, some concerns regarding the accessibility of pharmacists in this setting, as a strength of the traditional community pharmacist is both the convenient location and accessible working hours (Q15).

#### Isolated working patterns

A key finding was the perceived isolation of the dental team from other primary care health service providers. Although this is clearly an issue at a meso and macro level as described above, it also results in challenges for the management of individual patients and for individual practitioners. Participants reported poor collaborative care between medical and dental services, with limited interprofessional relationships and a distinct lack of formal referral pathways (Q16). As a result, participants reported that referrals to dental teams are typically informal and revolve around the principles of signposting patients to services, with little or no further follow-up.

The contextual, situational and geographical isolation of dental professionals reported at an individual level impacts on many aspects of patient care; in particular for dentists, obtaining accurate medication histories from patients and engaging with high quality collaborative patient care is a challenge (Q17).

#### Individual patient needs

Although wider policies and practices at a meso or macro level can impact on individual patient care, it was apparent that there are significant variations in individual patient needs and complexities. Consequently, healthcare practitioners often have to prioritise the management of certain conditions and/or the information that is provided to some patients. In many cases where patients have multiple co-morbidities, oral health was seen as having a lower priority and therefore was neglected in preference for matters deemed to be more clinically relevant to individuals by clinicians (Q18).

Although MRONJ is rare it can have significant negative effects on patients’ quality of life and the current lack of focus on preventive strategies at a population-based level is putting individual patients at risk. The interviews with patients highlighted the extent of the physical, psychological and social implications of the conditions that impact significantly on affected individuals. These include experiencing pain associated with MRONJ, regular need for analgesic medication, attendance at regular appointments with medical/dental professionals, concerns about potential complications and surgical management and social anxieties linked to eating and drinking in public (Q19, Q20). Greater focus on preventive care across this patient group could facilitate a reduction in the incidence of MRONJ.

### Functional and normative integration

#### Disconnection with general health

Oral health was seen by some participants as being the responsibility of dentists, with other healthcare professionals and patients not being aware of, or engaging with, the links between dental and general health. Education of both healthcare professionals and patients on the links to general health and well-being could serve to strengthen the relationship between oral and general healthcare (Q21).

#### Lack of shared clinical records

A significant barrier to effective collaborative oral healthcare is the current lack of access to shared medical records. Pharmacists have access to Summary Care Records, but currently GDPs have no access to either full or Summary Care Records. This was described by participants as a significant challenge that potentially impacts negatively on patient safety (Q22).

The safe prevention of MRONJ requires dental practitioners to be fully informed about a patient’s medication history and access to this information could improve patient safety, facilitating the implementation of MRONJ prevention guidelines. Dentists currently rely on patients to provide medication histories or need to contact general medical practices directly to obtain the information, which is a time-consuming and inefficient process. The access to shared records can also be a medico-legal issue from the perspective of medical/pharmacy professionals, as they have no record of what dental treatment has been performed or which medication may have been prescribed by GDPs (Q23).

#### Sub-optimal referral pathways

There is also a distinct lack of formalised referral pathways between dental and medical care settings, with participants reporting that referrals were typically informal and generally based on simply signposting patients (Q24). As a result, there is no follow-up of patients requiring referral, no records that referrals have taken place and minimal communication between care settings. This poses a significant challenge in the implementation of MRONJ prevention strategies, which require referrals from prescribers to dentists on prescribing of implicated medications and, in turn, assurance that patients are dentally fit to receive these medications before initiation. Formalised referral pathways, with shared medical records, could potentially facilitate the implementation of these recommendation and reduce the incidence of MRONJ in at-risk patients.

## Discussion

This study set out to explore the barriers and enablers for integrating oral healthcare and MRONJ prevention into pharmacists’ roles. The critical synthesis of data from five published papers and alignment to the Rainbow Model for Integrated Care provided strength and depth not achieved when each paper is viewed in isolation.

This research reveals that the contextual, situational and geographical isolation of dental teams is a barrier to optimal patient care. This is further enhanced by a lack of supportive and functionally integrative systems; such as no dental access to patient’s clinical information through full medical or Summary Care Records. Currently, dentists rely almost exclusively on patients to provide medical histories, posing a risk to patient safety; for example, evidence from research on osteoporosis patients highlighted a perceived lack of awareness of dentists about patients’ medical conditions with 46.5% of patients estimating that their dentist was unaware of their diagnosis of osteoporosis.^25^ Both dentists and physicians have expressed agreement that shared records could be useful in practice and the British Dental Association has previously highlighted that access to Summary Care Records could improve accuracy of medical histories and improve antimicrobial stewardship.^26,27^

The isolated knowledge base and perceived divisions between the medical and dental professions have been identified previously in research exploring the collaborative management of diabetes and identified as a significant contributing factor to mental illness and burnout amongst dentists.^28,29^ It was apparent in our work that there is limited communication between medical and dental services, with participants reporting a lack of formal referral pathways. This is similar to research exploring interprofessional collaboration in the context of management of diabetes, which found that dentists tend not to contact GPs regarding the management of patients and when they do so it is typically through the patient, as opposed to formal referral channels.^30^ Formalising and designating strategic work plans to develop interprofessional relationships and educational opportunities could potentially result in higher quality patient care and less isolation of oral health from general healthcare. On an individual, clinical level, the significant negative impact of MRONJ on quality of life has been illuminated through our research. The quality of life implications of MRONJ have been explored previously in the literature;^31^ however, the qualitative methodology adopted in our work has captured the significant negative impact of the condition from a human, rather than metric perspective, highlighting the ongoing challenges and the associated physical, psychological and social distress. These findings have substantively developed the evidence base that supports the preventive recommendations provided in currently established clinical guidelines.^12^ However, our research has highlighted that the preventive strategies recommended in published clinical guidelines are not being routinely followed in practice.^13–15^ This poses a question regarding the potential legal liability of the healthcare team if the recommended preventive care and associated patient education are not prioritised. The potential legal implications have previously been reported, including the finding that malpractice claims have been made in relation to MRONJ.^32^

The poor knowledge of oral health amongst pharmacists, GPs and patients was evident in this work and corresponds with findings reported in other studies;^9,21^ this is particularly apparent in relation to the crossover between oral health and general health and well-being, representing a focus for targeted educational initiatives. The introduction of interprofessional education in oral health could potentially facilitate an improved and reciprocal working knowledge that translates into increased implementation of preventive advice in relation to those medicines associated with MRONJ and the consequent provision of oral health advice to identifiably at-risk patients.

We have demonstrated that pharmacists can provide basic oral health interventions that produce positive intentions to change oral health behaviours among patients for whom oral health has not been a priority and who have a history of poor prior engagement with dental services. The proactive recruitment of patients to receive oral health interventions is a significant development in the evidence base, as studies to date have found that oral health advice is currently only provided by pharmacists in response to patient requests.^8^ The expansion of pharmacist’s roles to incorporate oral healthcare could facilitate an improvement in oral health in at-risk patient groups. The model designed and piloted in County Durham could potentially be transferable to other geographical settings and also represents a novel means of addressing oral health inequalities in populations that include poor dental attenders and those with high levels of oral healthcare intervention and support needs.^17^

### Strengths and limitations

This work is the cumulative synthesis of five individual papers. Adopting the Rainbow Model for Integrated Care as a theoretical framework provided both conceptual strength and depth to the research that is not possible to achieve when viewing each paper in abstraction from the other published work. All of the research was performed in the North East of England; a region with historically poor levels of oral health and challenging socio-economic demography. In keeping with high quality qualitative work, the research undertaken is not something from which the authors seek to generalise findings, but rather highlight transferable findings that can be considered in the context of similar locations and/or settings. Therefore, the authors fully acknowledge that the application of this work to other regional settings with differing demographics, or to other settings with differing dental and/or medical care infrastructure or service models, may not necessarily be possible. However, there is a clear need for change across the various levels of the system to support the integration of oral and general healthcare services.

### Implications for practice

At an organisational and professional level, greater consideration should be given to the incorporation of oral health into locally commissioned services, designed to meet local population needs. Services which require closer collaboration between the dental and medical professions could facilitate a reduction in isolated practices. Facilitators for functional integration, including the use of technology to support integrated working, shared medical records, and formal referral pathways, could improve optimal lines of communication and patient care.

Pharmacists have the potential to play a significant role in reducing oral health inequalities and increasing oral health educational opportunities with patients at high risk of complications and those with limited prior engagement with dental services. Interprofessional education not only has the potential to improve oral health knowledge, but could also improve understanding of professional roles, break down perceived disciplinary hierarchies and historically stereotyped roles, and build capacity and capability in the process. At a systems level there needs to be a greater focus by regulatory bodies to ensure integration of interprofessional education and inclusion of oral health education in the initial training of healthcare professionals and in professional practice.

There is significant need at a systems level to change the oral health attitudes and behaviours of the population. This could be facilitated through an NHS dental service that is clearly focused on prevention and through education of the general public on the benefits of good oral healthcare, specifically related to the relationships with general health and well-being. Collaborations between professional bodies and patient groups could serve to champion and role model optimal practice and encourage integration of care.

## Conclusion

Engaging all professional groups with the links between oral and general health could potentially facilitate the development of a collaborative mission to improve the quality and safety of patient care, and provide tangible benefits for both individual patients and the wider populations to which they belong. Our research has shown that pharmacists are currently underutilised in the provision of oral healthcare and in the prevention of MRONJ. Pharmacists, working across all settings, are a professional disciplinary healthcare group that could be utilised effectively to help address existing oral health inequalities with the ultimate aim of improving both the quality and safety of patient care in practice. This requires effective integration across micro, meso and macro levels of patient care, facilitated through education, supportive healthcare policies, shared clinical records and improved lines of communication.
